# Socioeconomic Factors Associated With Reports of Domestic Violence in Large Brazilian Cities

**DOI:** 10.3389/fpubh.2021.623185

**Published:** 2021-02-01

**Authors:** Marina Uchoa Lopes Pereira, Renato Simões Gaspar

**Affiliations:** ^1^Department of Public Health, Campinas State University (UNICAMP), Campinas, Brazil; ^2^Department of Public Health, Federal University of Maranhão (UFMA), São Luís, Brazil; ^3^School of Biological Sciences, Institute for Cardiovascular and Metabolic Research, University of Reading, Reading, United Kingdom

**Keywords:** domestic violence, mandatory reporting, racism, gender-based violence, Brazil

## Abstract

**Background:** Domestic violence is a traumatic experience that can lead to physical consequences, mental disorders and financial damage. Over 18 cases per 100,000 inhabitants were reported in Brazil between 2013 and 2014. The ministry of health poses a mandatory notification of all cases of domestic violence, which is essential, bearing in mind its systemic relation to various social issues and the extensive regional differences and high socioeconomic inequalities present in Brazil.

**Aim:** To analyze the characteristics of the notification rates of domestic violence and investigate the correlation of these with health and socioeconomic characteristics of large Brazilian cities.

**Methods:** Retrospective data on notifications of domestic violence was collected from the National Information System for Notifiable Diseases for Brazil, 2017. Dependent variables were collected from the Brazilian Institute of Geography and Statistics and Ministry of Citizenship. Inclusion criteria were: cities larger than 100.000 habitants and that had at least 20 reports, totaling 68.313 reports in 259 cities. These were stratified by age, race and sex of victim, type of violence used, violence perpetrator, place of occurrence and means of aggression. Proportional number of notified cases was calculated for each city to expose different characteristics of reports. A multiple linear regression model was used to investigate the correlation between report rates and different socioeconomic and health variables.

**Results:** The analysis showed a high proportion of repeated violence, use of body strength and over 50% were perpetrated by a partner or boyfriend. Report rates were higher for women, black individuals and children under four, highlighting subgroups of the population that were more vulnerable. Indeed, these groups were correlated differently with socioeconomic variables. Poverty, assessed as *Bolsa Fam*í*lia* investment, was correlated with domestic violence report rates across vulnerable groups.

**Conclusion:** The study showed that black women and children are more vulnerable to domestic violence, highlighting deleterious effects of patriarchy and structural racism within Brazilian society. Altogether, we suggest that reducing poverty, patriarchy and structural racism could lead to fewer cases of domestic violence.

## Introduction

Domestic or intra-family violence is defined as violence that happens between intimate partners or family members, especially at home, although not necessarily. The perpetrator of such violence can make use of various forms of aggression, such as: physical, psychological, sexual, financial violence and torture and it can be against children, the elderly, women or men (1). While many types of violence have been exposed and made visible, domestic violence often occurs out of sight due to the majority of cases happening at home, the vulnerability of the victim and historical and cultural aspects of modern societies (2, 3). Many of the victims are too young, too weak or too ill, therefore cannot protect themselves, thus making domestic violence a public health priority (3). Indeed, domestic violence is a traumatic experience that can lead to physical consequences, mental disorders and financial damage (4). It is traumatic not only to victims, but to nearby individuals exposed to it. For instance, the exposure to domestic violence during childhood has been associated with becoming an adult victim or perpetrator (5) and developing anxiety or trauma symptoms (6).

Given that domestic violence is a traumatic event, intensive research effort has been made to elucidate factors that may contribute to the occurrence of domestic violence—and therefore identify efficient ways to decrease cases of domestic violence. It is perceived that multiple aspects are involved, such as social-psychological (such as stress, family structure and the intergenerational transmission of violence) and cultural (such as patriarchy and racism) aspects (4). Although no racial, ethnic, or socio-economic group is immune (7), it is clear that domestic violence holds a systemic relation to various social issues. For instance, it has been reported a higher rates of domestic violence for non-white children (8), women (9), women with less schooling (10) and black and hispanic women (11).

From 2003 to 2012, in the United States, domestic violence accounted for 21% of all violent victimizations (12). Most of the available data on the prevalence of domestic violence is related to intimate partner violence, which is a type of domestic violence when the perpetrator is or was in an intimate relationship with the victim (i.e., boyfriend, girlfriend, spouse). Data from the World Health Organization indicate that up to 71% of women have been a victim of intimate partner violence (13). In parallel, a worldwide systematic review estimated that the lifetime prevalence of domestic violence varied from 1.9 to 70% amongst women (7). Notwithstanding, 22.6% of adults worldwide were reported to suffer physical abuse as a child, while 36.3% of adults experienced emotional abuse (14).

In Brazil, over 18 cases of domestic violence per 100,000 inhabitants were reported in the years of 2013 and 2014. This was over three times higher than what was observed in 2009 and 2010 (15). The theme gained more visibility after the creation, in 2006, of law number 11.340, known as the Maria da Penha Law (16). This legal instrument is focused on domestic violence against women and has an essential role in improving victim protection and aggressor punishment (16, 17), being considered one of the most advanced laws in the matter in modern legal systems (18).

Since it is often difficult to identify cases of domestic violence, due to its invisibility (4, 19), the Brazilian Unified Health System (SUS) poses a mandatory notification of suspected or confirmed cases of domestic violence. The notifications are filled by the healthcare personnel in Primary Health Care, which, in Brazil, consists of Family Health Strategy teams. These teams are composed of nurses, doctors, health technicians and community health agents, which are responsible for the care of specific families living in a particular territory (20). Family Health Strategy teams follow the attributes of longitudinality and integrality of care, which allow the teams to be more attentive to the territory and family context of the victims, resulting in a more comprehensive response to cases of violence (20, 21). The notification system in Brazil is centralized by the public database of the National Information System for Notifiable Diseases (SINAN), which was created in 2008 (22).

The notification of domestic violence is an important measure, given its association to social issues and the extensive regional differences and high socioeconomic inequalities present in Brazil (23). In this front, it has been shown that subgroups within the population are more vulnerable due to socio demographic characteristics, life context and lack of a consistent support network, both personal and institutional (24). This study aimed to characterize the notifications of domestic violence as a function of characteristics of both victim and perpetrator and to analyze the correlation between notification rates of domestic violence and health and socioeconomic characteristics of Brazilian cities. We therefore hypothesize that socioeconomic characteristics of cities as well as vulnerable groups of victims may be associated with higher notification rates of domestic violence in large Brazilian cities.

## Materials and Methods

### Data Sources and Variables

This is an observational retrospective study in which we used stratified sampling to analyze data notifications of domestic violence that occurred in the year of 2017 provided by the Brazilian Government through its national database, SINAN. Dependent variables used in correlation analyses were collected from the Brazilian Ministry of Health, the Brazilian Institute of Geography and Statistics (IBGE) and Ministry of citizenship. All data collected was from the year of 2017, except for population and demographic data, which were from 2010. This was due to the lack of estimates for population stratified by sex, race and age beyond the last census of 2010. Therefore, notification rates are likely overestimated in this study. All of the databases are public and free to access. Descriptive information and definition of variables are provided in Supplementary Table 1.

### Procedure

Domestic violence was the dependent variable used throughout the study and was defined as any type of violence perpetrated by: father, mother, stepfather, stepmother, brother, sister, partner/spouse, boyfriend, girlfriend, daughter or son; occurred at any site, but most likely at home. This definition is the same coined by Minayo (1).

In Brazil, the notification system is centralized by the ministry of health through the public database of the National Information System for Notifiable Diseases (SINAN). Currently 48 diseases or other incidents, including domestic violence, are of mandatory reporting. The notification forms must be filled by healthcare professionals in any health service, public or private and should contain sociodemographic data of the patient/victim and characteristics of the incident. In cases of violence, there are fields regarding information on the victim, the event, the aggressor and about the care provided. Those forms are sent to the municipal health department, where they are analyzed to feed the SINAN database, made public through TabNet, which is a tool of the Brazilian National Health System Information Technology Department (DATASUS). It is a public domain generic tabulator that allows anyone to generate information from the SINAN database (22, 25).

On the brazilian notification system, there are different rules for each disease or incident. When the incident is a violence, the healthcare professionals must report it whenever they suspect a violence is occurring, or whenever a victim has reported to suffer violence. Since it is not the role of healthcare professionals to investigate cases of violence, there is no follow up for notification purposes, only for improving the health of the victim. The information registered by the healthcare professional is confidential. It is not passed on to the police or publicly disclosed without going through health surveillance analysis of the municipal health department (22). Therefore, the report or notification of domestic violence was defined as any suspected case of domestic violence that has reached any health service of Brazil, which was notified by a healthcare professional.

### Statistical Methods

Notified cases of domestic violence were collected for cities with more than 100.000 habitants and that had more than 20 reports, totaling 68.313 reports in 259 cities. This was done to ensure data of higher quality and to decrease skewing of the data due to low number of reports. These were stratified by age, self-reported race and sex of the victim, as well as type of violence used, perpetrator of violence, place of occurrence and means of aggression. Proportional number of notified cases was calculated for each city. In order to calculate notification rates, the number of notified cases for each city was multiplied by 100,000 and divided by the population of that same city, according to the 2010 census. Data on notification rates as well as characteristics of the victim, aggressor, place of occurrence and means of violence used were analyzed through paired non-parametric Wilcoxon test for two variables (i.e., Sex), and Friedman test with Dunn's multiple comparison test for graphs with more than two variables, since data did not pass normality test. Bar graphs express mean ± S.E.M for 259 observations, comprehending 259 cities.

After initial assessment of which subgroups were reported to have higher notification rates, we conducted an ecological study to investigate the association of socioeconomic and health variables with the notification rate of domestic violence in vulnerable subgroups. Therefore, a multiple linear regression model was used. The model can be written as:

(1) $$\begin{array}{l} Y=\beta_{1}B+\beta_{2}C+\beta_{3}D+\beta_{4}E+\beta_{5}F+\beta_{6}G+\varepsilon \end{array}$$

Where *Y* is a single column vector of the report rate, *B* is Gross Domestic Product (GDP) per capita in R$ per 100.000 inhabitants, *C* is demographic density in person per km^2^, *D* is number of health places per 100.000 habitants, *E* is deaths due to assault per 100.000 inhabitants, *F* is *Bolsa Fam*í*lia* investment in R$, *G* is number of family health strategy teams per 100.000 inhabitants and ε is the error. All variables were log transformed, thus the coefficient should be interpreted as how a percentage change in x (dependent variables) affects the percentage change in y (notification of domestic violence). GraphPad Prism 8.0 software (GraphPad Software, San Diego, USA) was used for the statistical analysis.

### Ethics Statement

This research included data publicly available from the Brazilian Ministry of Health. The subjects' identities were not revealed to the researchers, and the data was obtained by healthcare professionals on duty, since the notification of domestic violence in health services is mandatory in Brazil. Therefore, written consent from the participants and ethical review and approval was not required for this study. This is in accordance with the Brazilian legislation and institutional requirements.

## Results

### Descriptive Characteristics of Domestic Violence Reports

There were 68,313 notified cases of domestic violence in large Brazilian cities in 2017. Table 1 shows descriptive statistics for these notifications according to region of notification, age, gender, race and schooling of the victim. The majority of reported cases were from the Southeast region, which is also the most populous part of Brazil. 23.31% of reports were for children between 0 and 4 years of age, whereas women accounted for over two thirds. In terms of race, 44.1% of notifications had a victim that was either black or *pardo* (black ethnicity). Most victims had <8 years of formal schooling. The report rate of domestic violence for each of the 259 cities included in this study is shown in Supplementary Table 2.

**Table 1:** Notified cases of domestic violence by region and socio-demographic characteristics of the victims. Brazil, 2017.

	**Notified cases**	
	***n***	**%**
**Region**		
North	2,964	4.3
Northeast	8,592	12.6
Southeast	36,891	54
South	14,875	21.8
Midwest	4,991	7.3
**Age (years)**		
0–4	15,926	23.31
5–9	6,433	9.42
10–14	7,523	11.01
15–19	6,560	9.60
20–29	9,734	14.25
30–39	8,840	12.94
40–49	5,427	7.94
50–59	2,828	4.14
≥60	4,986	7.30
Gender
Female	48,265	70.7
Male	20,018	29.3
**Race**		
White	26,502	38.8
Pardo^*^	24,486	35.8
Black	5,672	8.3
Indigenous/Asian	710	1.1
**Schooling (years)**		
<8	14,722	21.6
8–12	12,862	18.8
≥12	2,031	3
Brazil^**^	68,313	

* *Considered mix race*.

** *Cities with population >100,000 inhabitants and with more than 20 notifications of domestic violence in 2017*.

### Majority of Notified Cases of Domestic Violence Involved Physical Violence and Were Perpetrated by a Partner at Home Using Body Strength

**Figure 1:**
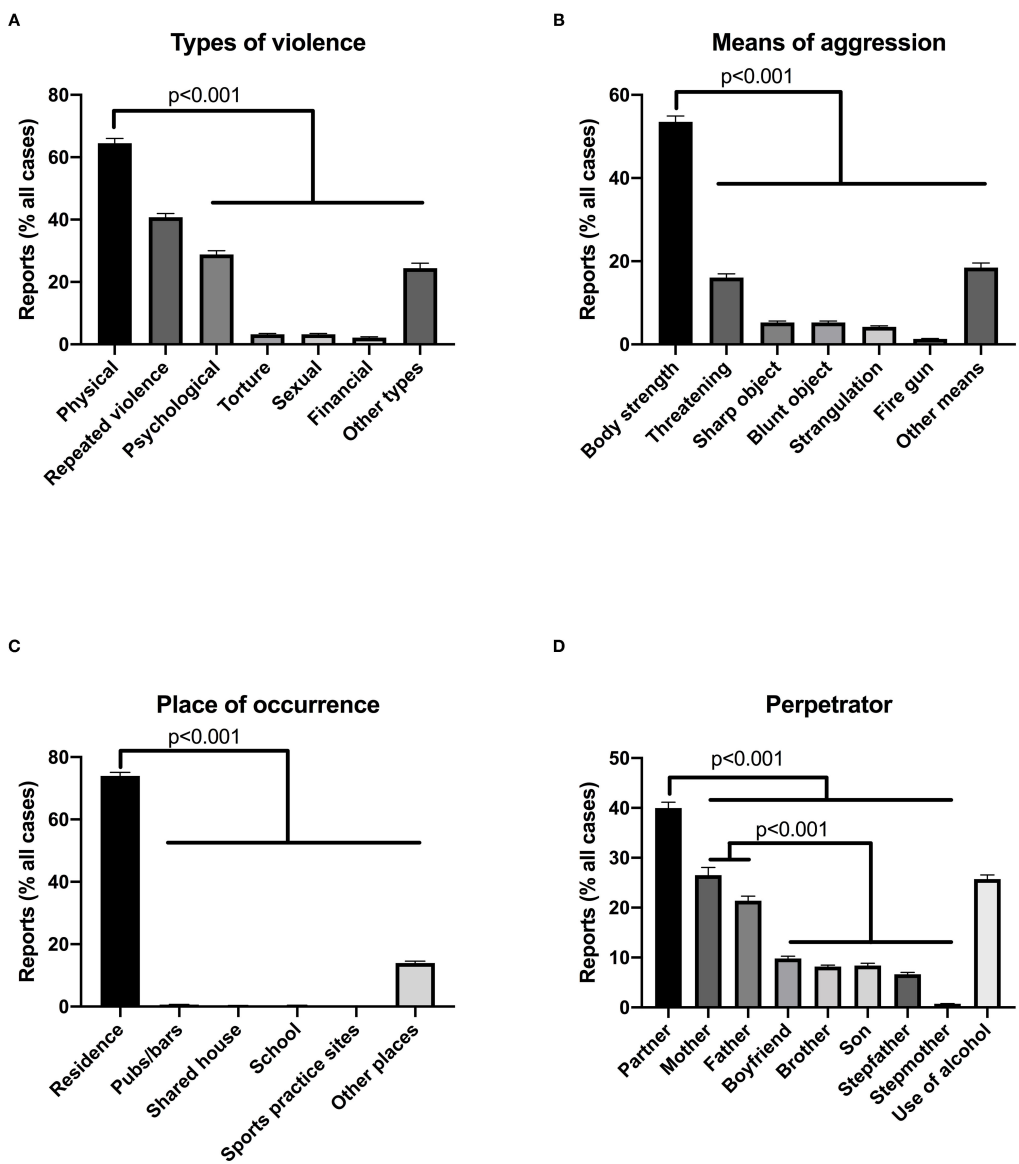
Characteristics of domestic violence reports in Brazilian cities with over 100.000 habitants, 2017. Reports were stratified by types of violence **(A)**, means of aggression **(B)**, place of occurrence **(C)** and data on perpetrator of the violence **(D)**. Proportional number of each subcategory was achieved after normalization to total number of cases. Of note, the sum of proportions can surpass 100%, since reports may involve more than one subcategory, for instance more than one perpetrator. Data analyzed through non-parametric Friedman test with Dunn's multiple comparison test, since data did not pass normality test. *P*-values shown in figure. Bar graphs express mean ± S.E.M for 259 observations.

There are several aspects entailing domestic violence, such as the type of violence used, means of aggression, place of occurrence and characteristics of the perpetrator. To examine the most common characteristics of notified cases, we conducted a paired analysis of the proportion of each subcategory of the above-mentioned aspects of domestic violence (Figure 1). Over 60% of reports involved physical violence, whilst 40% were repeated violence (Figure 1A), that is, the violence happened more than once. Accordingly, body strength was used in 53% of reports, being the most prevalent means of aggression (Figure 1B). Of note, only 1.3% of notified cases involved the use of a fire gun. Over 70% of cases occurred in residence (Figure 1C), as expected for reports of domestic violence. The partner was the most common perpetrator of violence, followed by parents (mother and father) (Figure 1D). However, if taken together, partner and boyfriend accounted for 50% of cases, whilst parents accounted for 47%. It is worth noting that notifications of domestic violence can involve more than one type of violence, means of aggression or perpetrator, thus making the sum of the proportions higher than 100%. This, however, does not preclude the analysis of the most common characteristics of reports.

### Higher Domestic Violence Notification Rate for Women, Black Individuals and Children

**Figure 2:**
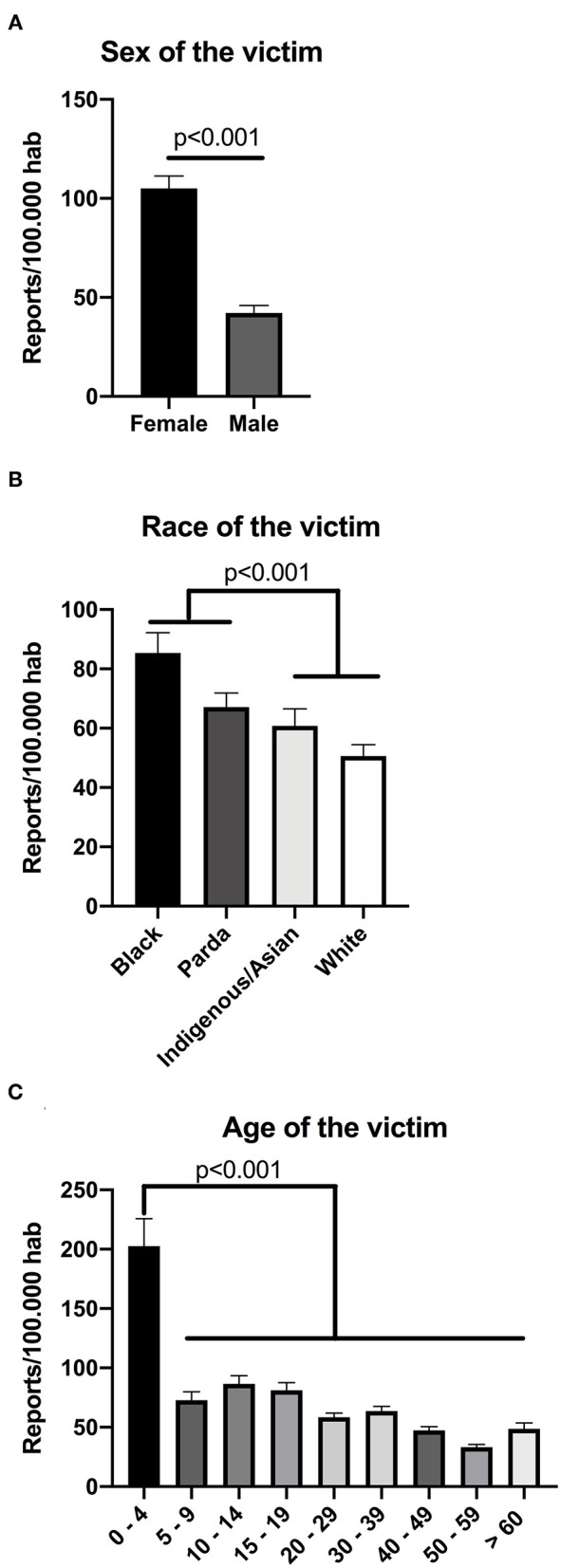
Domestic violence report rates according to victim's sex, race and age. Brazil, 2017. Report rates were calculated according to each subcategory of population within cities. These were divided by sex **(A)**, race **(B)** or age **(C)** of the victim. Data in **(A)** analyzed through paired non-parametric Wilcoxon test, whereas data in **(B)** and **(C)** were analyzed through non-parametric Friedman test with Dunn's multiple comparison test, since data did not pass normality test. *P*-values shown in figure. Bar graphs express mean ± S.E.M for 259 observations.

To analyze aspects of the victims, we calculated the report rates of domestic violence for different subgroups of the population for each of the 259 cities analyzed. As exhibited in Figure 2, there were twice as many notified cases for women than men, reaching over 100 cases per 100,000 inhabitants. Similarly, there were 60% more cases in black individuals than white ones (Figure 2B). The highest report rate was observed in children up to 4 years of age, which reached 200 cases per 100,000 inhabitants - almost 4 times higher than the report rate for other age brackets (Figure 2C). We have also analyzed race and age in men and women separately (Supplementary Figure 1). From this, we observed that black women were disfavored when compared to white women, whilst the contrast was less evident for men (Supplementary Figures 1A,B). Similar patterns and report rates were observed for both men and women when stratified by age (Supplementary Figures 1C,D). Overall, these data indicate that black women and children up to 4 years of age presented more report rates for domestic violence in Brazil.

### Socioeconomic Factors Correlated With Report Rates of Domestic Violence

In order to examine whether report rates for domestic violence were correlated with socioeconomic factors, we performed multiple linear regression models (Table 2). These were run for total report rates as well as for women, black population and children up to 4 years, which were the subgroups that presented higher report rates according to Figure 2. Sensitivity analysis for the total regression, in which a stepwise addition of variables was performed, is presented in Supplementary Table 3 and suggests that correlations in Table 2 are robust. GDP per capita was positively correlated with report rates for total population (0.24% change per 1% change in notification rate, 95%CI 0.007 to 0.48), as well as for women (0.28% change per 1% change in notification rate, 95%CI 0.06 to 0.51), whereas *Bolsa Fam*í*lia* investment was inversely correlated for total population, women and black individuals. Interestingly, the strength of the association between *Bolsa Fam*í*lia* investment and notification rates was similar for the total population, women and black individuals (around −0.15% change per 1% change in notification rate). This indicates that richer cities tended to have higher report rates for domestic violence and poorer cities tended to have lower report rates. Curiously, there was an inverse correlation between domestic violence notification rates for women and number of health places (−0.18% change per 1% change in notification rate, 95%CI −0.35 to −0.001). Finally, notifications in children were positively associated with demographic density, which was not present in other population groups. Thus, it is likely that different subgroups within the population are affected differently by socioeconomic factors.

**Table 2:** Multiple linear regression of possible determinants of domestic violence. Brazil, 2017.

	**Groups at higher risk**							
**Social determinants**	**Notification rate total**		**Notification rate women**		**Notification rate Black population**		**Notification rate Children 0 to 4 y.o**.	
	**Coefficient (95% CI)**	***P*-value**	**Coefficient (95% CI)**	***P*-value**	**Coefficient (95% CI)**	***P*-value**	**Coefficient (95% CI)**	***P*-value**
GDP per capita (1.000 R$/inhabitant)	0.245 (0.007 to 0.483)	**0.043**	0.289 (0.061 to 0.517)	**0.013**	0.204 (−0.101 to 0.509)	0.189	0.372 (−0.021 to 0.767)	0.0641
Demographic density (person per km^2^)^a^	−0.017 (−0.089 to 0.054)	0.632	−0.049 (−0.118 to 0.019)	0.1614	0.001 (−0.089 to 0.092)	0.973	0.130 (0.012 to 0.249)	**0.030**
Health places per 100.000 inhabitants	−0.129 (−0.315 to 0.055)	0.169	−0.179 (−0.357 to −0.001)	**0.0477**	0.048 (−0.184 to 0.280)	0.684	−0.138 (−0.444 to 0.167)	0.373
Deaths due to assault per 100.000 inhabitants	−0.019 (−0.181 to 0.143)	0.818	−0.026 (−0.182 to 0.130)	0.742	−0.195 (−0.401 to 0.010)	0.0626	0.198 (−0.067 to 0.464)	0.142
*Bolsa Família* invested (R$)	−0.132 (−0.252 to −0.011)	**0.031**	0.156 (−0.271 to −0.041)	**0.007**	−0.183 (−0.333 to −0.032)	**0.0171**	−0.084 (−0.279 to 0.111)	0.395
Family health strategy teams per 100.000 inhabitants	0.017 (−0.233 to 0.269)	0.888	−0.002 (−0.243 to 0.238)	0.982	0.036 (−0.278 to 0.351)	0.819	0.029 (−0.383 to 0.441)	0.889

*All variables were included in the model and log transformed. therefore coefficient should be interpreted as how a percentage change in x (dependent variables) affects percentage change in y (notification of domestic violence). CI, Confidence interval. p < 0.05 are in bold*.

## Discussion

Data herein presented underlie associated factors of domestic violence reports in large Brazilian cities in the last year available, 2017. We show that there was a high proportion of repeated violence (i.e., that occurred more than once), that the majority of cases involved physical violence with the use of body strength and that over 50% were perpetrated by a partner or boyfriend. There were higher rates for women, black individuals and children under four, highlighting subgroups of the population that were more vulnerable. Indeed, these groups were correlated differently with socioeconomic variables. Poverty, assessed as *Bolsa Fam*í*lia* investment, was negatively correlated with domestic violence report rates across vulnerable groups, that is, poorer cities tended to present higher rates of domestic violence notifications. Thus, we show that black women and children are more vulnerable to domestic violence and suggest that improving household size, reducing poverty and structural racism would be an effective way to reduce cases of domestic violence.

Women accounted for 70% of the victims of reports and had a report rate two times higher than men. This, coupled with a high proportion of partners, boyfriends and fathers as perpetrators of the violence indicates a historical relation of power between men and women. This reinforces the deleterious impact of patriarchy within the Brazilian society, since it has been shown that patriarchy is systematic and institutionalized in western culture (26–28). This rationale may also apply to the high report rates found in children, given the dominance aspect of patriarchy is extended to children and adolescents (26) and considering fathers, stepfathers and brothers are perpetrators who benefit from this historical relation of power. Although it has been reported a higher prevalence of child maltreatment by mothers (29), fathers are more commonly perpetrators of sexual violence (30) and severe physical aggression, such as head injury and fractures (29).

Because the perpetrators are often family members, these have close contact to the victims, as well as a power-based relationship. A reoccurrence of the violence would be expected (2). This highlights a complex and well-established victim-aggressor dynamic, which facilitates the silencing of the victim and the invisibility of the violence (2, 3, 28). Hence, it is imperative to typify this form of violence in the legal system and strengthen report systems to increase visibility.

The most common means of aggression and type of violence were body strength and physical violence, which are in consonance with the literature (2, 28). Physically injured victims are more inclined to look for health services, when compared to other means of violence, such as sexual, psychological or financial (28). This, coupled with the minimal use of fire guns, indicates that the perpetrator indeed maintains a relationship of power toward the victims without the need of further means of aggression (2).

We found a positive correlation between GDP per capita and report rates in women and total population. It is possible that richer cities have more structured health systems (31) that are more prepared to identify and report cases of domestic violence. Alternatively, it has been consistently shown that economic development measured as increases in GDP per capita increases income inequality in both developing and developed countries (32). Income inequality has been strongly correlated with rates of intimate partner violence in Latin American countries (33). Thus, GDP per capita may influence report rates of domestic violence either due to it being associated with more developed health systems or this might be an indirect effect of rises in income inequality and consequent increase of intimate partner violence.

Since we were unable to measure income inequality at city-level, we used *Bolsa Fam*í*lia* investment as a proxy for poverty, given that this cash-transfer programme targets families below the poverty line. From this analysis, we found a negative correlation between *Bolsa Fam*í*lia* investment and report rates in women, black population and total, meaning that cities with less poverty report more cases of domestic violence. The lack of correlation between GDP per capita and report rates in black individuals may suggest this population does not access city-level economic growth. This reinforces the idea of a social hierarchy in which black individuals (and within these, black women) are at the bottom of the social hierarchy (34, 35). Moreover, these results indicate poorer cities report fewer cases of domestic violence. We suggest that efforts to mitigate structural racism could be an efficient way to reduce cases of domestic violence given the high report rates found for the black population.

Furthermore, we found that demographic density had a positive correlation with report rates in children. In regards to household size, more densely packed houses can generate a more stressful environment, leading to a higher occurrence of violence amongst relatives. This may be influenced by the way households are organized in Brazil, considering that mothers were also frequent perpetrators of violence. It has been observed a decrease in the number of couples with children and an increase in the proportion of households composed of single mothers with children (36, 37). Therefore, since there has been an increase in Brazilian mothers cohabiting with their children, one could expect an increase in the number of domestic violence perpetrated by mothers.

The number of family health strategy teams, which are part of primary healthcare, were not associated with the number of reports in any of the groups. This is counter-intuitive, since these teams are part of the reporting system for domestic violence. One possible explanation may be that while the professionals could prevent some domestic violence incidents, especially their reoccurrence, they could also identify cases more readily, increasing the number of notifications. Additionally, this result could be an indicative of fragilities and obstacles within primary health care in Brazil. Indeed, it has been shown that there are training gaps, lack of intersectoral communication and difficulties in recognizing domestic violence by primary health care teams (20). Nonetheless, primary health care has a pivotal role in identifying cases of domestic violence, since professionals have knowledge of the community and should therefore be familiar with the victims (20). Strengthening primary health care is therefore essential and raising awareness of health professionals toward gender violence, racism and child maltreatment should be of paramount importance to reduce cases of domestic violence.

This study has several limitations that we would like to point out. We expect a considerable number of underreporting due to above mentioned issues, namely power-relationship between perpetrator and victim, awareness of primary health care professionals and lack of structured reporting systems. In addition, it is possible to have errors in the notification forms, caused by the difficulty of filling combined with the lack of awareness of the professionals who fill them out. To try and minimize the impact of these limitations, we only used data from the last year available and only from large cities, which should have more structured report systems. Despite this, we reinforce the importance of carrying out studies using official notification data to comprehend the magnitude of the problem and create evidence for better policy makers decisions. We also point out the need of continuing education so that professionals are better prepared to identify and report cases correctly.

Here we showed that domestic violence is a complex issue within the Brazilian society. A high proportion of these reports were of repeated violence (i.e., that occurred more than once), involved physical violence and were perpetrated by a partner or boyfriend. There were higher report rates for children under four, women and black individuals, emphasizing subgroups of the population that were more vulnerable. This highlights deleterious effects of patriarchy and structural racism within the Brazilian society. Domestic violence report rates across vulnerable groups were associated with poverty, assessed as *Bolsa Fam*í*lia* investment. Altogether, we suggest that women, black individuals and children are more vulnerable to domestic violence. These data reinforce previous evidence that show that domestic violence is a type of gender-based violence with racial implications (11). Therefore, future studies should explore if the reduction of poverty, patriarchy and/or structural racism could be an effective strategy to reduce cases of domestic violence.

## Data Availability Statement

Data is available upon reasonable request to the corresponding author.

## Author Contributions

MP and RG designed the study, analyzed data, drafted the manuscript, reviewed, and approved the final version of the manuscript. All authors contributed to the article and approved the submitted version.

## Conflict of Interest

The authors declare that the research was conducted in the absence of any commercial or financial relationships that could be construed as a potential conflict of interest.
