# Supplementary material for: Socioeconomic Factors Associated With Reports of Domestic Violence in Large Brazilian Cities
Source: Front Public Health. 2021 Feb 1;9:623185. doi: 10.3389/fpubh.2021.623185 (PMC7884961; doi:10.3389/fpubh.2021.623185)
Supplement: Supplementary file 1 [file Table_1.DOCX]

**Supplementary table 1. Descriptive information and sources of data used in the study.**

| **Variable name** | **Unit** | **Source** | **Definition** |
| --- | --- | --- | --- |
| Notification rate of domestic violence | Notification per 100.000 habitants | National Information System for Notifiable Diseases (SINAN), 2017 | Number of notifications for domestic violence divided by population number multiplied by 100.000 |
| GDP per capita | Reais (R$) per 100.000 habitants | Brazilian Institute of Geography and Statistics (IBGE), 2017 | Gross domestic product divided by midyear population multiplied by 100.000. GDP is the sum of gross value added by all resident producers in the economy plus any product taxes and minus any subsidies not included in the value of the products. It is calculated without making deductions for depreciation of fabricated assets or for depletion and degradation of natural resources |
| Demographic Density | Person per km^2^ | IBGE 2010 Census | Number of people resident in a given city divided by the area of the city |
| Number of health places per 100.000 habitants | Number per 100.000 | Brazilian Ministry of Health – National Registry of Health Facilities (CNES), Dec 2017 | Health places consist of any establishment that conducts health promotion, prevention or treatment of diseases. These include, but are not limited to: hospitals, primary health care units, pharmacies, emergency units, etc |
| Deaths due to assault per 100.000 habitants | Deaths per 1,000 habitants | Brazilian Ministry of Health, 2017 | Deaths due to external causes related to assault according to the International Classification of Diseases (ICD) 10: X85 – X99 and Y00 – Y09. These were collected for population between 5 and 74 years of age in the year of 2017 |
| *Bolsa Familia* investment | Reais (R$) | Brazilian Ministry of Citizenship, 2017 | Transfer of resources for the *Bolsa Familia* programme from the Federal government to cities in Reais (R$). *Bolsa Familia* is a cash transfer programme directed to families below the poverty line (income lower than R$ 89,00 per person per month). It can be used as a proxy of poverty |
