# Supplementary material for: Socioeconomic Factors Associated With Reports of Domestic Violence in Large Brazilian Cities
Source: Front Public Health. 2021 Feb 1;9:623185. doi: 10.3389/fpubh.2021.623185 (PMC7884961; doi:10.3389/fpubh.2021.623185)
Supplement: Supplementary file 2 [file Table_2.DOCX]

**Supplementary Table 2. Rate of notified cases for domestic violence per city. Brazil, 2017.**

| City | Notified cases (n) | Notification rate/ 100,000 inhabitants |
| --- | --- | --- |
| **North** |  |  |
| Rio Branco (AC) | 173 | 45.12 |
| Manaus (AM) | 736 | 34.55 |
| Macapá (AP) | 32 | 6.74 |
| Santana (AP) | 71 | 61.49 |
| Abaetetuba (PA) | 34 | 22.17 |
| Altamira (PA) | 146 | 131.02 |
| Ananindeua (PA) | 87 | 16.86 |
| Barcarena (PA) | 21 | 17.33 |
| Belém (PA) | 363 | 25.00 |
| Marabá (PA) | 31 | 11.41 |
| Parauapebas (PA) | 54 | 26.69 |
| Tailândia (PA) | 73 | 70.65 |
| Tucuruí (PA) | 195 | 176.45 |
| Porto Velho (RO) | 156 | 30.03 |
| Ji-Paraná (RO) | 22 | 16.58 |
| Ariquemes (RO) | 26 | 24.22 |
| Boa Vista (RR) | 103 | 31.02 |
| Araguaína (TO) | 233 | 132.42 |
| Palmas (TO) | 408 | 142.27 |
| **Northeast** |  |  |
| Arapiraca (AL) | 23 | 9.82 |
| Maceió (AL) | 273 | 26.53 |
| Camaçari (BA) | 39 | 13.14 |
| Eunápolis (BA) | 72 | 62.45 |
| Feira de Santana (BA) | 420 | 66.93 |
| Itabuna (BA) | 24 | 10.86 |
| Jequié (BA) | 22 | 13.56 |
| Juazeiro (BA) | 193 | 87.03 |
| Lauro de Freitas (BA) | 27 | 13.66 |
| Salvador (BA) | 714 | 24.17 |
| Vitória da Conquista (BA) | 121 | 34.70 |
| Caucaia (CE) | 62 | 17.12 |
| Fortaleza (CE) | 713 | 27.14 |
| Juazeiro do Norte (CE) | 24 | 8.88 |
| Maranguape (CE) | 37 | 29.25 |
| Sobral (CE) | 138 | 67.14 |
| Caxias (MA) | 66 | 40.58 |
| Codó (MA) | 24 | 19.87 |
| Imperatriz (MA) | 30 | 11.78 |
| São José de Ribamar (MA) | 49 | 27.77 |
| São Luís (MA) | 245 | 22.44 |
| Timon (MA) | 32 | 19.09 |
| Campina Grande (PB) | 70 | 17.06 |
| João Pessoa (PB) | 178 | 21.93 |
| Santa Rita (PB) | 28 | 20.46 |
| Cabo de Santo Agostinho (PE) | 122 | 59.61 |
| Camaragibe (PE) | 157 | 100.41 |
| Caruaru (PE) | 69 | 19.38 |
| Garanhuns (PE) | 321 | 231.53 |
| Igarassu (PE) | 37 | 32.06 |
| Jaboatão dos Guararapes (PE) | 406 | 58.34 |
| Olinda (PE) | 221 | 56.55 |
| Paulista (PE) | 101 | 30.76 |
| Petrolina (PE) | 1,224 | 356.62 |
| Recife (PE) | 854 | 52.27 |
| Santa Cruz do Capibaribe (PE) | 31 | 29.31 |
| São Lourenço da Mata (PE) | 356 | 317.58 |
| Vitória de Santo Antão (PE) | 320 | 232.60 |
| Parnaíba (PI) | 38 | 25.24 |
| Teresina (PI) | 269 | 31.64 |
| Mossoró (RN) | 31 | 10.49 |
| Natal (RN) | 109 | 12.31 |
| Parnamirim (RN) | 120 | 47.11 |
| Aracaju (SE) | 162 | 24.92 |
| Nossa Senhora do Socorro (SE) | 20 | 10.99 |
| **Southeast** |  |  |
| Cachoeiro de Itapemirim (ES) | 45 | 21.26 |
| Cariacica (ES) | 115 | 29.69 |
| Colatina (ES) | 100 | 80.31 |
| Guarapari (ES) | 83 | 67.39 |
| Linhares (ES) | 37 | 21.89 |
| São Mateus (ES) | 45 | 35.03 |
| Serra (ES) | 430 | 85.55 |
| Vila Velha (ES) | 110 | 22.62 |
| Vitória (ES) | 604 | 166.33 |
| Araguari (MG) | 21 | 17.88 |
| Araxá (MG) | 119 | 114.11 |
| Barbacena (MG) | 89 | 65.11 |
| Belo Horizonte (MG) | 679 | 26.90 |
| Betim (MG) | 417 | 97.62 |
| Conselheiro Lafaiete (MG) | 247 | 193.92 |
| Contagem (MG) | 99 | 15.03 |
| Coronel Fabriciano (MG) | 25 | 22.66 |
| Divinópolis (MG) | 46 | 19.58 |
| Governador Valadares (MG) | 83 | 29.55 |
| Ibirité (MG) | 47 | 26.48 |
| Ipatinga (MG) | 125 | 47.86 |
| Itabira (MG) | 131 | 109.82 |
| Ituiutaba (MG) | 121 | 115.76 |
| Juiz de Fora (MG) | 53 | 9.40 |
| Lavras (MG) | 23 | 22.52 |
| Montes Claros (MG) | 56 | 13.93 |
| Muriaé (MG) | 152 | 140.04 |
| Passos (MG) | 30 | 26.21 |
| Patos de Minas (MG) | 33 | 21.87 |
| Poços de Caldas (MG) | 119 | 71.65 |
| Pouso Alegre (MG) | 76 | 51.65 |
| Ribeirão das Neves (MG) | 44 | 13.38 |
| Sabará (MG) | 45 | 33.10 |
| Santa Luzia (MG) | 152 | 69.44 |
| Sete Lagoas (MG) | 59 | 24.98 |
| Teófilo Otoni (MG) | 22 | 15.50 |
| Uberaba (MG) | 230 | 70.06 |
| Uberlândia (MG) | 163 | 24.09 |
| Varginha (MG) | 106 | 78.89 |
| Vespasiano (MG) | 39 | 31.87 |
| Angra dos Reis (RJ) | 192 | 98.65 |
| Barra Mansa (RJ) | 73 | 40.68 |
| Belford Roxo (RJ) | 360 | 72.61 |
| Cabo Frio (RJ) | 22 | 10.18 |
| Campos dos Goytacazes (RJ) | 23 | 4.69 |
| Duque de Caxias (RJ) | 628 | 70.48 |
| Itaboraí (RJ) | 54 | 23.24 |
| Japeri (RJ) | 159 | 157.06 |
| Macaé (RJ) | 670 | 274.43 |
| Magé (RJ) | 272 | 114.56 |
| Mesquita (RJ) | 111 | 64.81 |
| Nilópolis (RJ) | 51 | 32.21 |
| Niterói (RJ) | 212 | 42.48 |
| Nova Friburgo (RJ) | 84 | 45.31 |
| Nova Iguaçu (RJ) | 1,784 | 223.38 |
| Petrópolis (RJ) | 71 | 23.81 |
| Queimados (RJ) | 128 | 88.04 |
| Resende (RJ) | 70 | 55.15 |
| Rio das Ostras (RJ) | 121 | 85.74 |
| Rio de Janeiro (RJ) | 6,147 | 94.28 |
| São Gonçalo (RJ) | 173 | 16.48 |
| São João de Meriti (RJ) | 126 | 27.36 |
| Volta Redonda (RJ) | 81 | 30.54 |
| Americana (SP) | 147 | 62.86 |
| Araçatuba (SP) | 41 | 21.04 |
| Araraquara (SP) | 263 | 113.97 |
| Araras (SP) | 41 | 31.23 |
| Atibaia (SP) | 26 | 18.61 |
| Barueri (SP) | 121 | 45.23 |
| Bauru (SP) | 41 | 11.03 |
| Birigui (SP) | 39 | 32.31 |
| Botucatu (SP) | 327 | 229.40 |
| Bragança Paulista (SP) | 79 | 48.12 |
| Campinas (SP) | 1,038 | 87.79 |
| Caraguatatuba (SP) | 145 | 124.16 |
| Carapicuíba (SP) | 47 | 11.85 |
| Catanduva (SP) | 65 | 53.86 |
| Cotia (SP) | 84 | 35.33 |
| Cubatão (SP) | 39 | 30.29 |
| Diadema (SP) | 322 | 77.06 |
| Embu das Artes (SP) | 128 | 47.93 |
| Ferraz de Vasconcelos (SP) | 98 | 51.89 |
| Franca (SP) | 70 | 20.16 |
| Francisco Morato (SP) | 86 | 50.12 |
| Franco da Rocha (SP) | 49 | 32.78 |
| Guarujá (SP) | 27 | 8.56 |
| Guarulhos (SP) | 595 | 44.10 |
| Hortolândia (SP) | 196 | 88.21 |
| Indaiatuba (SP) | 42 | 17.53 |
| Itapecerica da Serra (SP) | 46 | 26.91 |
| Itapetininga (SP) | 43 | 26.86 |
| Itaquaquecetuba (SP) | 448 | 124.22 |
| Jacareí (SP) | 255 | 110.94 |
| Jundiaí (SP) | 209 | 51.04 |
| Limeira (SP) | 63 | 20.94 |
| Marília (SP) | 162 | 68.87 |
| Mauá (SP) | 554 | 119.91 |
| Mogi das Cruzes (SP) | 732 | 168.70 |
| Osasco (SP) | 148 | 21.21 |
| Ourinhos (SP) | 67 | 59.92 |
| Paulínia (SP) | 61 | 59.51 |
| Pindamonhangaba (SP) | 109 | 66.46 |
| Piracicaba (SP) | 72 | 18.12 |
| Poá (SP) | 41 | 35.50 |
| Praia Grande (SP) | 22 | 7.10 |
| Presidente Prudente (SP) | 53 | 23.53 |
| Ribeirão Pires (SP) | 54 | 44.32 |
| Ribeirão Preto (SP) | 126 | 18.47 |
| Rio Claro (SP) | 192 | 94.60 |
| Santa Bárbara d'Oeste (SP) | 83 | 43.25 |
| Santana de Parnaíba (SP) | 153 | 116.01 |
| Santo André (SP) | 305 | 42.64 |
| Santos (SP) | 263 | 60.50 |
| São Bernardo do Campo (SP) | 900 | 108.77 |
| São Caetano do Sul (SP) | 55 | 34.46 |
| São Carlos (SP) | 64 | 26.01 |
| São José do Rio Preto (SP) | 852 | 189.06 |
| São José dos Campos (SP) | 1,170 | 166.38 |
| São Paulo (SP) | 7,677 | 63.41 |
| São Vicente (SP) | 77 | 21.37 |
| Sertãozinho (SP) | 196 | 159.81 |
| Sorocaba (SP) | 97 | 14.70 |
| Sumaré (SP) | 123 | 45.05 |
| Suzano (SP) | 125 | 42.99 |
| Taboão da Serra (SP) | 96 | 34.33 |
| Taubaté (SP) | 144 | 46.76 |
| Várzea Paulista (SP) | 66 | 55.50 |
| **South** |  |  |
| Almirante Tamandaré (PR) | 418 | 362.33 |
| Apucarana (PR) | 177 | 133.39 |
| Araucária (PR) | 241 | 175.33 |
| Cambé (PR) | 47 | 44.61 |
| Campo Largo (PR) | 61 | 47.91 |
| Cascavel (PR) | 602 | 188.36 |
| Colombo (PR) | 205 | 86.35 |
| Curitiba (PR) | 4,427 | 231.98 |
| Foz do Iguaçu (PR) | 284 | 107.56 |
| Guarapuava (PR) | 134 | 74.29 |
| Londrina (PR) | 597 | 106.91 |
| Maringá (PR) | 347 | 85.32 |
| Paranaguá (PR) | 57 | 37.26 |
| Pinhais (PR) | 205 | 158.37 |
| Piraquara (PR) | 344 | 319.25 |
| Ponta Grossa (PR) | 92 | 26.72 |
| São José dos Pinhais (PR) | 417 | 135.60 |
| Toledo (PR) | 64 | 47.22 |
| Alvorada (RS) | 128 | 61.49 |
| Bagé (RS) | 62 | 50.73 |
| Bento Gonçalves (RS) | 234 | 203.36 |
| Cachoeirinha (RS) | 125 | 98.18 |
| Canoas (RS) | 222 | 64.56 |
| Caxias do Sul (RS) | 649 | 134.26 |
| Erechim (RS) | 51 | 49.31 |
| Gravataí (RS) | 86 | 31.26 |
| Passo Fundo (RS) | 435 | 218.81 |
| Pelotas (RS) | 29 | 8.42 |
| Porto Alegre (RS) | 885 | 59.60 |
| Rio Grande (RS) | 288 | 137.55 |
| Santa Cruz do Sul (RS) | 32 | 25.11 |
| Santa Maria (RS) | 104 | 37.35 |
| São Leopoldo (RS) | 62 | 26.85 |
| Sapucaia do Sul (RS) | 32 | 22.94 |
| Uruguaiana (RS) | 371 | 285.86 |
| Viamão (RS) | 76 | 29.95 |
| Balneário Camboriú (SC) | 105 | 77.62 |
| Blumenau (SC) | 135 | 38.74 |
| Brusque (SC) | 93 | 72.19 |
| Chapecó (SC) | 63 | 29.54 |
| Criciúma (SC) | 330 | 156.13 |
| Florianópolis (SC) | 230 | 47.34 |
| Itajaí (SC) | 509 | 239.40 |
| Jaraguá do Sul (SC) | 39 | 22.83 |
| Joinville (SC) | 473 | 81.96 |
| Lages (SC) | 42 | 26.50 |
| Palhoça (SC) | 66 | 40.02 |
| São José (SC) | 161 | 67.16 |
| Tubarão (SC) | 39 | 37.34 |
| **Midwest** |  |  |
| Brasília (DF) | 890 | 29.28 |
| Águas Lindas de Goiás (GO) | 69 | 35.24 |
| Anápolis (GO) | 188 | 50.11 |
| Aparecida de Goiânia (GO) | 199 | 36.71 |
| Formosa (GO) | 26 | 22.45 |
| Goiânia (GO) | 346 | 23.60 |
| Luziânia (GO) | 79 | 39.58 |
| Novo Gama (GO) | 38 | 34.52 |
| Rio Verde (GO) | 88 | 40.54 |
| Senador Canedo (GO) | 22 | 20.86 |
| Trindade (GO) | 42 | 34.63 |
| Valparaíso de Goiás (GO) | 95 | 59.56 |
| Campo Grande (MS) | 2,036 | 232.90 |
| Corumbá (MS) | 539 | 490.45 |
| Dourados (MS) | 73 | 33.48 |
| Três Lagoas (MS) | 50 | 42.56 |
| Cuiabá (MT) | 91 | 15.42 |
| Rondonópolis (MT) | 37 | 16.64 |
| Sinop (MT) | 83 | 61.09 |
| **Total** | 68,313 |  |
