# Supplementary material for: Socioeconomic Factors Associated With Reports of Domestic Violence in Large Brazilian Cities
Source: Front Public Health. 2021 Feb 1;9:623185. doi: 10.3389/fpubh.2021.623185 (PMC7884961; doi:10.3389/fpubh.2021.623185)
Supplement: Supplementary file 3 [file Table_3.DOCX]

Supplementary Table 3. Sensitivity analysis of multivariate regression model for total number of notified cases. Brazil, 2017.

|  | Social determinants of Domestic Violence notification amongst groups at higher risk | | | | | | | | | | | |
| --- | --- | --- | --- | --- | --- | --- | --- | --- | --- | --- | --- | --- |
|  | **Notifications –**  **total** | | **Notifications –**  **total** | | **Notifications –**  **total** | | **Notifications –**  **total** | | **Notifications –**  **total** | | **Notifications –**  **total** | |
|  | Coefficient (SE) | P-value | Coefficient (SE) | P-value | Coefficient (SE) | P-value | Coefficient (SE) | P-value | Coefficient (SE) | P-value | Coefficient (SE) | P-value |
| GDP per capita (1.000 R$/inhabitant) | 0.219  (0.095) | **0.022** | 0.223  (0.095) | **0.020** | 0.261  (0.111) | **0.019** | 0.236  (0.113) | **0.038** | 0.235  (0.112) | **0.037** | 0.245  (0.120) | **0.043** |
| Demographic density (person per km^2^) ^a^ |  |  | -0.030  (0.032) | 0.343 | -0.038  (0.034) | 0.265 | -0.045  (0.034) | 0.196 | -0.020  (0.036) | 0.571 | -0.017  (0.036) | 0.632 |
| Health places per 100.000 inhabitants |  |  |  |  | -0.057  (0.086) | 0.505 | -0.084  (0.090) | 0.350 | -0.108  (0.090) | 0.229 | -0.129  (0.094) | 0.169 |
| Deaths due to assault per 100.000 inhabitants |  |  |  |  |  |  | -0.077  (0.072) | 0.286 | -0.004  (0.079) | 0.958 | -0.019  (0.082) | 0.818 |
| Bolsa familia invested (R$) |  |  |  |  |  |  |  |  | -0.133  (0.060) | **0.028** | -0.132  (0.060) | **0.031** |
| Family health strategy teams per 100.000 inhabitants |  |  |  |  |  |  |  |  |  |  | 0.017  (0.127) | 0.888 |

All variables were included in the model and log transformed. therefore coefficient should be interpreted as how a percentage change in x (dependent variables) affects percentage change in y (notification of domestic violence). SE: Standard Error. p<0.05 are in bold.

Model 1: only GDP per capita as independent variable

Model 2: GDP and demographic density as independent variables

Model 3: GDP, demographic density and number of health places as independent variables

Model 4: GDP, demographic density, health places and deaths due to assault as independent variables

Model 5: GDP, demographic density, health places, deaths due to assault and Bolsa Familia investment as independent variables

Model 6: GDP, demographic density, health places, deaths due to assault, Bolsa Familia investment and number of family health strategy teams as independent variables
