# Supplementary figures and images for: Socioeconomic Factors Associated With Reports of Domestic Violence in Large Brazilian Cities
Source: Front Public Health. 2021 Feb 1;9:623185. doi: 10.3389/fpubh.2021.623185 (PMC7884961; doi:10.3389/fpubh.2021.623185)

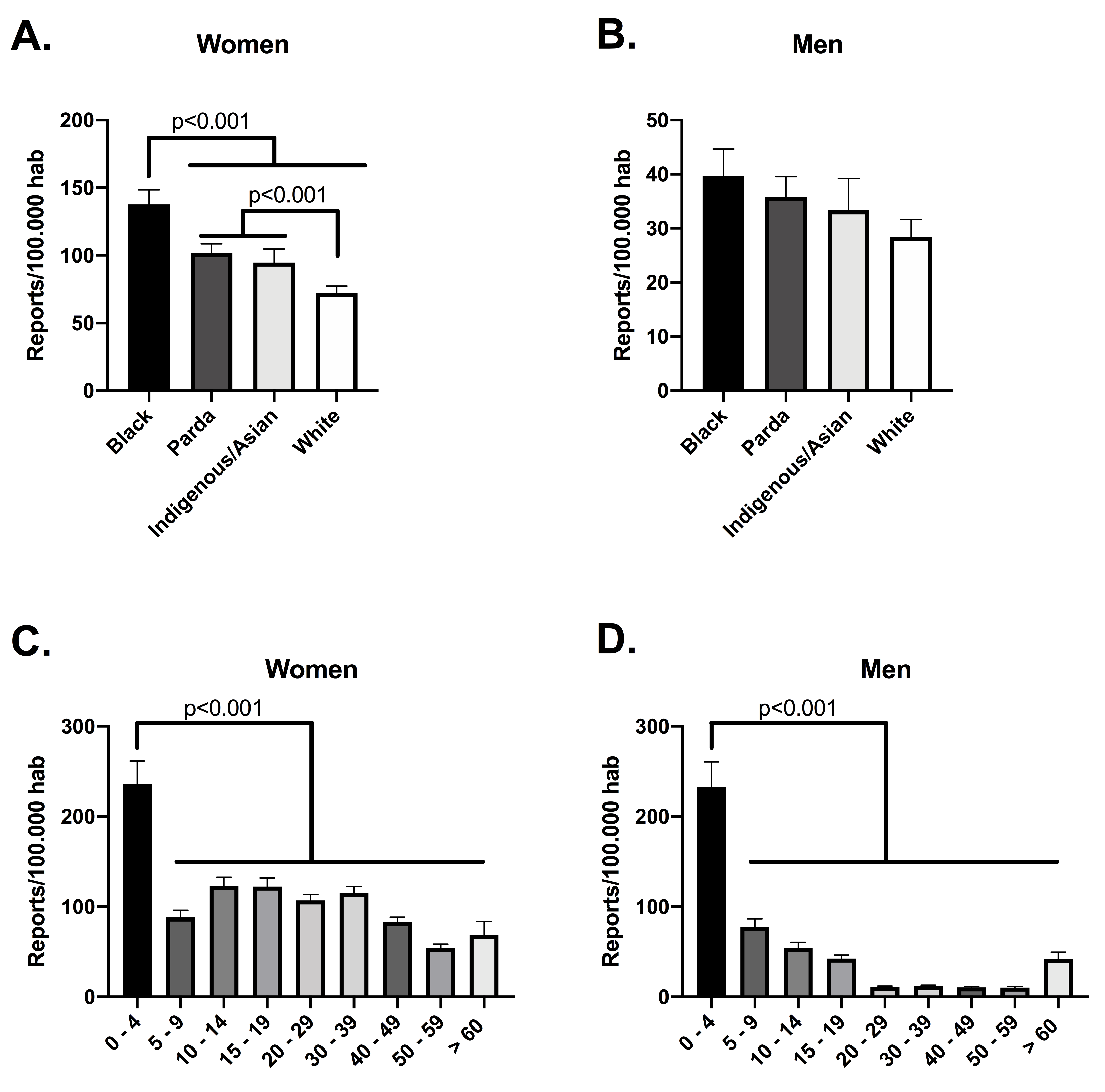

Supplement: Supplementary file 4 [file Image_1.TIFF]
